# An integrated framework for optimal infrastructure planning for decarbonising heating

**DOI:** 10.1016/j.mex.2023.102184

**Published:** 2023-04-15

**Authors:** Pooya Hoseinpoori, Jeremy Woods, Nilay Shah

**Affiliations:** a Centre for Environmental Policy (CEP), Imperial College London, United Kingdom; b Department of Chemical Engineering, Imperial College London, United Kingdom; c Centre for Process Systems Engineering (CPSE), Imperial College London, United Kingdom; d Sustainable Gas Institute (SGI), Imperial College London, United Kingdom

**Keywords:** Energy system modelling, Decarbonising heating, Infrastructure planning, Gas network modelling, Electricity system modelling, Hydrogen, Electrification, Optimization, HEGIT (Heat, Electricity and Gas Infrastructure and Technology)

## Abstract

This paper presents the HEGIT (Heat, Electricity and Gas Infrastructure and Technology) model for optimal infrastructure planning for decarbonising heating in buildings. HEGIT is an optimisation model based on Mixed Integer Linear Programming. The model co-optimises the integrated operation and capacity expansion planning of electricity and gas grids as well as heating technologies on the consumer side while maintaining the security of supply and subject to different environmental, operational and system-wide constraints. The three main features of the HEGIT model are:

• It incorporates an integrated unit commitment and capacity expansion problem for coordinated operation and long-term investment planning of the electricity and gas grids.
• It incorporates the flexible operation of heating technologies in buildings and demand response in operation and long-term investment planning of gas and electricity grids.
• It incorporates a multi-scale techno-economic representation of heating technologies design features into the whole energy system modelling and capacity planning.

These features enable the model to quantify the impacts of different policies regarding decarbonising heating in buildings on the operation and long-term planning of electricity and gas grids, identify the cost-optimal use of available resources and technologies and identify strategies for maximising synergies between system planning goals and minimising trade-offs. Moreover, the multi-scale feature of the model allows for multi-scale system engineering analysis of decarbonising heating, including system-informed heating technology design, identifying optimal operational setups at the consumer end, and assessing trade-offs between consumer investment in heating technologies and infrastructure requirements in different heat decarbonisation pathways.

## Graphical abstract

**Figure:**
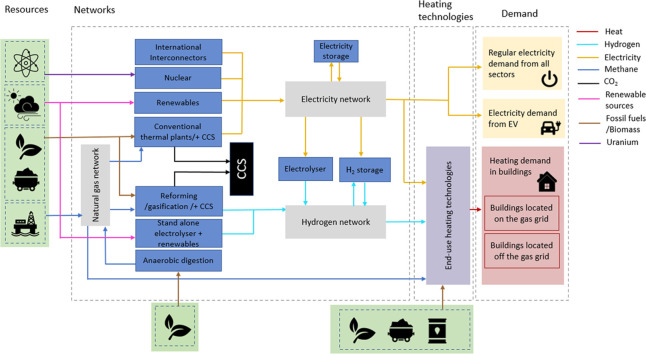

## Nomenclature

**Sets**
a: Planning periods [year]
bt: Type of biomass [-]
c: Days of each year [day]
gb: Type of gas boilers gb⊂K
h: Heating demand category
hp: Type of heat pump hp⊂K
i: Technologies in the electricity grid, i∈I [-]
ib: Electricity generating technologies the use biomass as fuel, ib⊂i [-]
ic: Conventional electricity generating technologies, ic⊂i [-]
ig: Electricity generating technologies, ig⊂i [-]
im: Electricity generating technologies the use Methane as fuel, im⊂i [-]
ir: Variable renewable technologies, ir⊂i [-]
is: Electricity storage technologies in the electricity grid, is⊂i [-]
j: Gas production/storage technologies j∈J [-]
jb: Hydrogen production technologies that use biomass as fuel, jb⊂jp [-]
jc: biomethane production technologies jc∈J [-]
je: Hydrogen production technologies supplied by the electricity grid, je⊂jp [-]
jf: Hydrogen production technologies that use fossil fuels as fuel, jf⊂jp [-]
jl: Linepack storage, jl⊂js [-]
jm: Hydrogen production technologies that use methane as fuel, jm⊂jp [-]
jp: Hydrogen production technologies, jp⊂j [-]
jr: Renewable hydrogen production technologies, jr⊂jp [-]
js: Hydrogen storage technologies, js⊂j [-]
jus: Underground hydrogen storage, jus⊂js [-]
k: All the heating technologies at the consumer side k∈K [-]
l: Alternative low carbon energy vectors l∈L [-]
nv: Energy vectors that will not be replaced, nv⊂V [-]
rv: Energy vectors that will be replaced, rv⊂V [-]
t: Time periods [hour]
v: Energy vectors used for heating, v∈V [-]
z: All the technologies, z=i∪j∪k [-]
zg: All the generation technologies in electricity and gas grids, zg=ig∪js [-]
zn: All the technologies in electricity and gas grids, zn=i∪j [-]
zs: All the storage technologies in electricity and gas grids, zs=is∪js [-]

**Parameters & Variables**
$X_{ini}\left(z\right)$: Number of available units of technology z at the beginning of the horizon [-]
Δa: step width planning years [year]
ΔT: Weighted average temperature increase required at consumer-side [°C]
$\delta_{eff}\left(h,v,a\right)$: Demand reduction by energy efficiency improvement for energy vector v for demand group h at year a [MWh]
$\eta_{gb}$: Gas boiler efficiency
γ(zg): Efficiency of generation technologies zg [-]
λ(h,v,a): Conversion efficiency for each energy vector v for each demand group h at year a [-]
μ(zs): Round trip efficiency of storage unit zs
ρ: Density of water [kg/L]
AV(ir,c,t): Availability factor of technology ir in day c and time t [% MW ]
b(z,a): Number of new built units of technology z in year a [-]
Br(z): Build rate of technology z [unit/year]
$C_{p}$: Specific heating capacity of water [J/kgK]
$C_{dcg}$: Cost of decommissioning gas grid [£/MWh]
CAC: Cost of avoided ${\text{CO}}_{2}$ [£$/{\text{tonne}}_{{\text{CO}}_{2}}$]
$Cap_{UK}$: Maximum available capacity for each salt cavern formation in the UK [MWh]
CAPEX(zn): Capital expenditure of technology zn [£/MW]
CI(v,a): Carbon intensity of the energy vector v at year a [${\text{tonne}}_{{\text{CO}}_{2}}/\text{MWh}$]
CM: Capacity margin [% MW]
COP(hp,c,t): Coefficient of performance for heat pump type hp [-]
$cost_{bms}\left(a\right)$: Total annual cost of biomass required in year a [£]
Ctx(a): Carbon price floor in year a [£$/{\text{tonne}}_{{\text{CO}}_{2}}$]
$pr_{impElec}$: Price of imported electricity [£/MWh]
Ctx(a): Carbon price floor in year a [£$/{\text{tonne}}_{{\text{CO}}_{2}}$]
$d_{bms}\left(a\right)$: Total annual biomass demand in year a [MWh]
$d_{BM}\left(a,c,t\right)$: Demand for biomethane [MWh]
$D_{elec}\left(a\right)$: Regular annual electricity demand projection at year a [MWh]
$D_{EV}\left(a\right)$: Annual electricity demand projection for EV at year a [MWh]
$d_{ev}\left(h,v,a\right)$: Demand for primary energy vector v used directly for supplying demand group h at year a [MWh]
$d_{e}\left(a,c,t\right)$: Total electricity demand [MWh]
$d_{g}\left(gb,a,c,t\right)$: Demand for gas from boiler gb [MWh]
$d_{H_{2}}\left(a\right)$: Total annual demand for hydrogen at year a [MWh]
$d_{NG}\left(a,c,t\right)$: Demand for natural gas [MWh]
Disc(a): Discount factor in year a [£]
$DP_{elec}\left(c,t\right)$: Normalised demand profile for regular electricity [-]
$DP_{EV}\left(c,t\right)$: Normalised demand profile for EV charging [-]
$DP_{heat}\left(c,t\right)$: Normalised demand profile for heating in buildings [-]
DT(zg): Minimum down time requirements of technology zg [hour]
$Ems_{heat}\left(a\right)$: Emission target for heating in buildings at year a [${\text{tonne}}_{{\text{CO}}_{2}}$]
EUC: Total investment on fuel switching over the planning horizon [£]
$F_{inMax}\left(jus\right)$: The maximum injection rate to each underground storage unit [MW]
$F_{outMax}\left(jus\right)$: The maximum discharging rate from each underground storage unit [MW]
hd(a): Average heating demand from each household in year a [MWh]
$imp_{NG}\left(a\right)$: Natural gas import in year a [MWh]
INJ: Injection rate to salt caverns as % of working gas
Ins(k): Installation cost share for heating heating technology k [-]
$IO_{NG}\left(a\right)$: Industrial and other demand sources for natural gas in year a [£]
$LP_{Max}$: Maximun linepack state of charge per unit of demand [${\text{MWh}}_{s}/{\text{MWh}}_{d}$]
$LP_{Min}$: Minimum linepack state of charge per unit of demand [${\text{MWh}}_{s}/{\text{MWh}}_{d}$]
LT(z): Lifetime of technology z [year]
$LT_{ini}\left(z\right)$: Lifetime of available units of technology z [year]
$m_{in}\left(k,a,c,t\right)$: Water inflow to the tank integrated with heating technology k [kg]
$m_{out}\left(k,a,c,t\right)$: Water outflow from tank integrated with heating technology k to demand [kg]
$m_{tank}\left(k,a,c,t\right)$: Hot water stored in the tank integrated with heating technology k [kg]
$Max_{bms}\left(bt\right)$: Maximum available biomass of type bt [MWh]
Mtn(k): Maintenance cost for heating technology k [£]
n(zn,a,c,t): Number of units of technology zn [-]
NC(zg): Nominal capacity of technology zg [MW]
$NC_{HP}\left(a\right)$: Nominal thermal capacity of heat pump installed in year a [kW]
$NC_{s}\left(jus\right)$: Storage capacity of underground gas storage technologies jus [MWh]
NL: Gas network losses [£]
NRF: Network reinforcement factor for transmission and distribution networks
o(zs,a,c,t): Number of units of storage technology zs [-]
OM: Operating margin requirement in the gas grid
OPC(zn,a): Operational costs of technology zn in year a [£/MWh]
OPEX(a): Total operational costs in year a [£]
OPEXF(zn): Fixed operational costs of technology zn in a year [£/MWh]
OPEXSU(zn): Start-up costs of technology zn [£/MWh]
p4gs(a,c,t): Electricity demand for gas storage in year a day c time t [MWh]
$p4H_{2}\left(a,c,t\right)$: Electricity demand for hydrogen production in year a day c time t [MWh]
$p_{bh}\left(a,c,t\right)$: Electricity demand from backup heater in year a day c time t [MWh]
$p_{e}\left(ig,a,c,t\right)$: Electricity generation from technology ig [MWh]
$p_{H_{2}}\left(jp,a,c,t\right)$: Hydrogen production from technology jp [MWh]
$p_{hp}\left(hp,a,c,t\right)$: Electricity to heat pump hp [MWh]
$P_{NG}\left(a\right)$: Domestic production of natural gas at year a [MWh]
$pd_{e}\left(ig,a,c,t\right)$: Electricity to demand from technology ig [MWh]
$pd_{H_{2}}\left(jp,a,c,t\right)$: Hydrogen to demand from generation technology jp [MWh]
$pis_{e}\left(is,a,c,t\right)$: Electricity to storage technology is [MWh]
$PL_{e}\left(a\right)$: Peak electricity load in year a [MWh]
$PL_{H_{2}}\left(a\right)$: Peak load of hydrogen demand in year a [MW]
$Pr_{bms}\left(bt\right)$: Cost of biomass type bt [£/MWh]
$pr_{impElec}$: Price of imported electricity [£/MWh]
$ps_{e}\left(ig,a,c,t\right)$: Electricity to grid-level storage from technology ig [MWh]
$ps_{H_{2}}\left(js,a,c,t\right)$: Hydrogen input to storage technology js [MWh]
$pst_{H_{2}}\left(jp,a,c,t\right)$: Hydrogen to storage from generation technology jp [MWh]
$q_{backup}\left(k,a,c,t\right)$: Heat output from backup heater integrated with heating technology k [MWh]
$q_{d}\left(h,v,a\right)$: Heat demand supplied by energy vector v for demand group h at year a [MWh]
$q_{lc}\left(l,h,v,a\right)$: Heating demand supplied by alternative low carbon energy vectors l replacing energy vector v for demand group h at year a [MWh]
$q_{out}\left(k,a,c,t\right)$: Heat output from the thermal storage tank for each heating technology k [MWh]
$q_{s}\left(h,v,a\right)$: Heat delivered by energy vector v to demand group h at year a [MWh]
$r_{e}\left(ig,a,c,t\right)$: Reserve capacity provided by technology ig [MWh]
RD(zg): Maximum ramp down rate of technology zg [%]
Res: Minimum days of reserve requirement [day]
RM: Absolute reserve margin [% MW]
RU(zg): Maximum ramp up rate of technology zg [%]
$S_{Cush}\left(jus\right)$: Minimum storage inventory level of underground storage-cushion gas [% MWh]
$s_{e}\left(is,a,c,t\right)$: Effective state of charge of technology is [MWh]
$s_{H_{2}}\left(js,a,c,t\right)$: Inventory level in storage technology js [MWh]
$S_{Max}\left(is\right)$: Maximum storage inventory level [% MW]
$S_{Min}\left(is\right)$: Minimum storage inventory level [% MW]
$sd_{e}\left(is,a,c,t\right)$: Electricity from storage to demand from technology is [MWh]
$sd_{H_{2}}\left(js,a,c,t\right)$: Hydrogen to demand from storage technology js [MWh]
SE(a): Electricity system emission target in year a [${\text{tonne}}_{{\text{CO}}_{2}}$]
SI: Minimum system inertia demand [MWs]
$sl_{H_{2}}\left(jl,a,c,t\right)$: Hydrogen from underground storage units to Linepack [MWh]
SM: Supply margin requirement in the gas grid
$sr_{e}\left(is,a,c,t\right)$: Reserve capacity provided by technology is [MWh]
$stl_{H_{2}}\left(jus,a,c,t\right)$: Hydrogen from storage unit jus to linepack i [MWh]
$su_{bms}\left(bt,a\right)$: Supply of biomass type bt in year a [MWh]
SWDI: Shanon Weiner Diversity Index
$T_{air}\left(a,c,t\right)$: Air temperature [°C]
$T_{soil}\left(a,c,t\right)$: Soil temperature [°C]
TE(i,*): Features of technology i, where * is: (various)
  PminMinimumoutput%MW,
  PmaxMaximumoutput%MW,
  $Ems\;\;\;\mathrm{Emission}\;\text{rate}\;\;\;\;{\text{tonne}}_{{\text{CO}}_{2}}/{\text{MWh}}_{H_{2}}$,
  CmaxMaximumcapacityprovision%MW,
  RPReservepotential,abilityfactortoprovidereservecapacity%MW,
  IPInertiaprovisionpotentialMws/MW
TF(j,*): Features of technology j, where * is: (various)
  PminMinimumoutput%MW,
  PmaxMaximumoutput%MW,
  $Ems\;\;\;\mathrm{Emission}\;\text{rate}\;\;\;\;{\text{tonne}}_{{\text{CO}}_{2}}/{\text{MWh}}_{H_{2}}$
TL: Losses in transmission network [%]
TSC: Total system cost [£]
TSE: Total system emission [${\text{tonne}}_{{\text{CO}}_{2}}$]
TSE: Total system emission at year a [${\text{tonne}}_{{\text{CO}}_{2}}$]
u(zg,a,c,t): Number of units of technology zg [-]
UC(gb): Unit cost of gas boiler gb [£]
$ug_{H_{2}}\left(a,c,t\right)$: Unmet Hydrogen demand-gas shedding [MWh]
$up_{d}\left(a,c,t\right)$: Unmet electricity demand-load shedding [MWh]
UT(zg): Minimum up time requirements of technology zg [hour]
$V_{tank}$: Hot water cylinder storage capacity integrated with technology k [L]
$VoLL_{e}$: Value of Lost electricity load [£/MWh]
$VoLL_{g}$: Value of Lost heat Load [£/MWh]
w(zg,a,c,t): Number of units of technology zg shutting down [-]
WD: Withdrawal rate from salt caverns as % of working gas
WF(c): Representative day weighting factor [-]
WFA: Annual weighting factor [-]
WR: Dynamic reserve for wind electricity generation [% MW]
x(z,a): Number of units of technology z operational in year a, cumulative [-]
$e_{BM}\left(jc\right)$: Emission rate of biomethane production from anaerobic digestion [$t_{{\text{CO}}_{2}}/\text{MWh}$]

Specifications table

**Table:** 

Subject area:	Energy system modelling
More specific subject area:	System modelling for decarbonising heating
Name of your method:	HEGIT (Heat, Electricity and Gas Infrastructure and Technology)
Name and reference of original method:	[1]: Clara F. Heuberger. (2017). *Electricity Systems Optimisation with capacity eXpansion and Endogenous technology Learning (ESO-XEL)*. Zenodo. https://doi.org/10.5281/zenodo.1048943
Resource availability:	The data used in HEGIT model for the case study of the UK is available at https://doi.org/10.1016/j.enconman.2022.115952

### Introduction

Energy system models are integrated frameworks that provide quantitative insights into alternative energy systems. They provide structured stories about future developments through scenario analysis and based on an organised exploration of data and assumptions [2]. Whole energy system models have played a major role in informing energy policy and shaping the energy strategy discourse over the past two decades. As decision support tools, they have been widely used to explore the solution space for decarbonisation and provide insights into the costs, feasibility, path dependency, and energy security implications of possible transition pathways to a low carbon energy system [3].

Achieving climate change mitigation targets will require decarbonisation efforts across all sectors of the economy, particularly the energy sector. Decarbonising heating in buildings is central to this energy transition challenge. Heat is one of the largest energy-consuming sectors and a major source of emissions in many countries with a cold climate. The scale of the heat challenge differs significantly from state to state depending on various factors, such as climate conditions, building stock, energy prices, the heating technology portfolio mix in buildings and the current structure of the energy system in a country. There is a wide range of options for decarbonising heating, with different pathways leading to very different energy systems. Modelling the future heat system is complex, and an effective representation of different heat decarbonisation pathways requires capturing the interactions across many domains, including buildings, heating systems, the electricity sector, and the existing fuel supply infrastructure such as the gas and electricity grids [[3], [4]].

This paper presents the HEGIT (Heat, Electricity and Gas Infrastructure and Technology) model for optimal infrastructure planning for decarbonising heating in buildings. HEGIT is a multi-scale integrated electricity and gas systems’ unit commitment, economic dispatch and capacity expansion planning optimisation model based on Mixed Integer Linear Programming (MILP). The model is designed to investigate the coordinated transition of electricity and gas grids and assess the impacts of different policies and decisions for decarbonising heating in buildings on the operation and long-term investment planning of these networks. HEGIT is implemented in GAMS and uses CPLEX as the mixed integer linear programming solver. The model co-optimises the short-term operations and long-term investment planning of electricity and gas grids as well as the individual heating technologies in buildings while ensuring the security of supply and subject to different environmental, operational and system-wide constraints. The main outputs of the model are the portfolio mix and cost-optimal operation schedule of heating technologies in buildings, the cost-optimal dispatch schedules, the optimal technology mix and the evolution over the planning horizon for both gas and electricity grids. Such a coordinated approach is crucial for understanding the interaction between major components of the system, exploring cross-system solutions and identifying trade-offs between infrastructure requirements and investments in heating technologies at the consumer side for decarbonising heating. It also provides insights into the necessary levels of deployment of different options, the likely levels of investment required, key periods when strategic decisions need to be made, and the system-wide impacts of different choices [3].

**Fig. 1:**
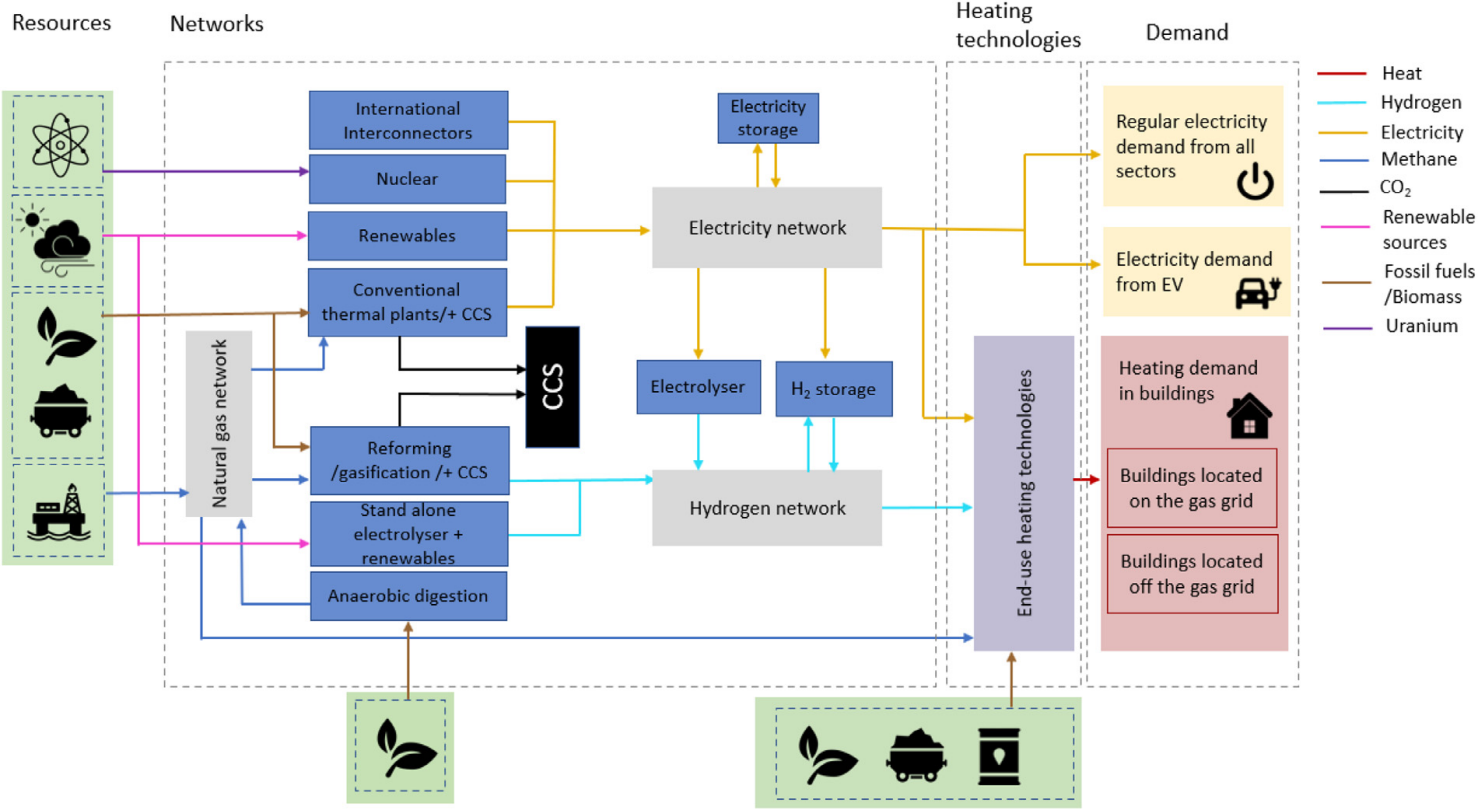
HEGIT framework structure. The centrepiece of HEGIT is a MILP, multi-scale unit commitment and capacity planning model of integrated gas and electricity systems. The framework has four main parts that are combined into a single optimisation framework [7]: (i) demand, (ii) heating technologies, (iii) networks and (iv) resources. The graph is adapted from the contribution of Hoseinpoori et al. in Ref [7].

**Fig. 2:**
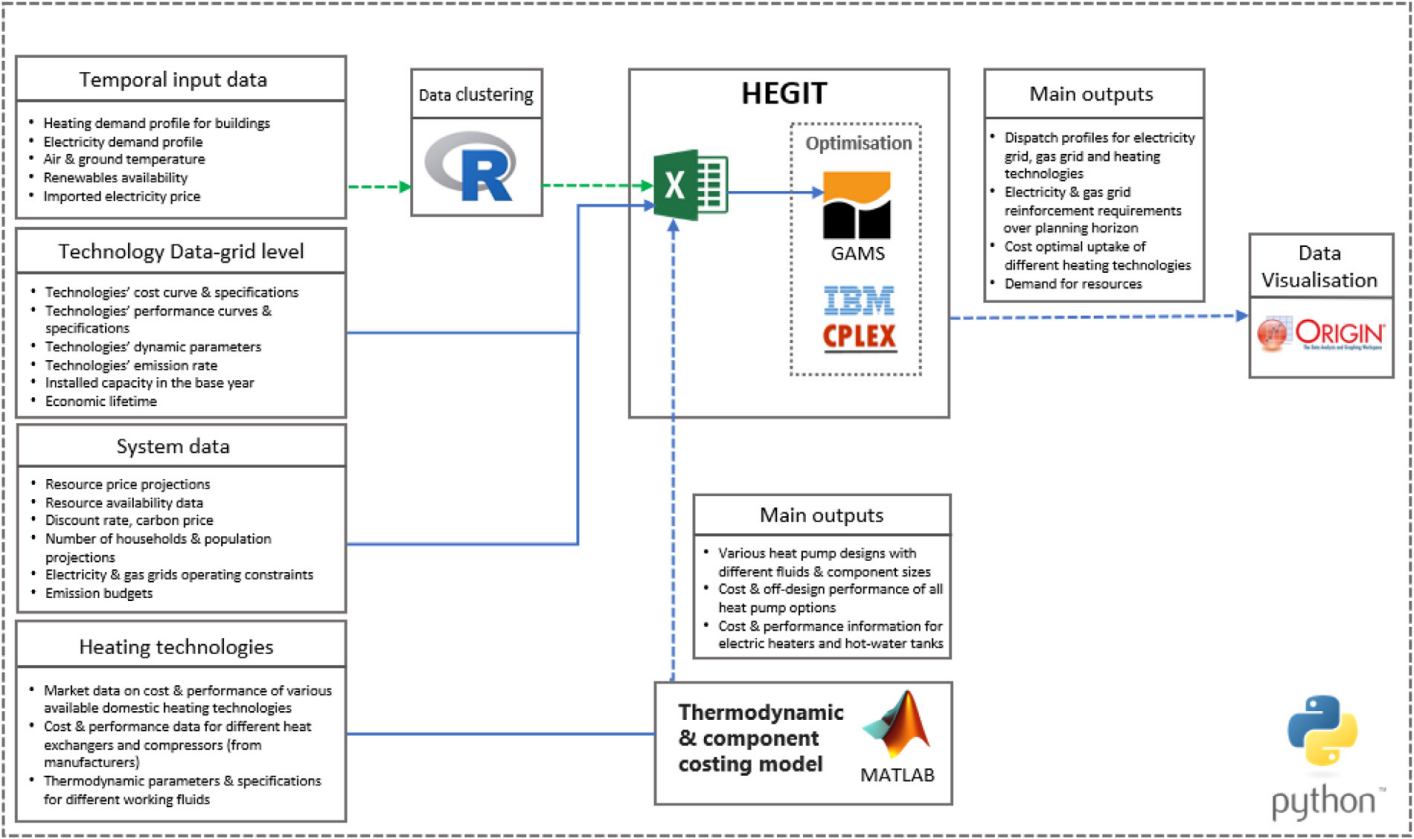
Schematic of the data flow, modelling and software integration in the HEGIT framework.

**Fig. 3:**
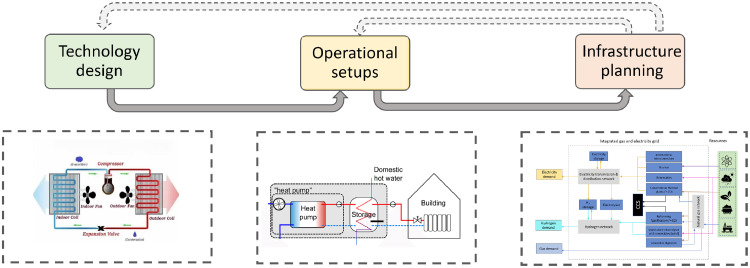
Multi-scale system engineering analysis using HEGIT for system-informed heating technology design, identifying optimal operational setups at the consumer end, and assessing trade-offs between consumer investment in heating technologies and infrastructure requirements in different heat decarbonisation pathways.

To have a more accurate representation of heating technologies’ cost and performance, such as variations in heat pump performance with outside temperature, and to study the impacts of different heat pump designs features on the operation and planning of the electricity grid, we soft-linked HEGIT with the thermodynamic and component-costing models developed by Olympios et al. [5] that capture the variations in cost and performance of heating options such as heat pumps. The cost and performance equations of heating technologies were derived from the thermodynamic and component cost models developed at Imperial College London’s Clean Energy Processes laboratory (CEP). These cost and performance curves are validated using the cost and performance characteristics of different technologies collected from an in-depth analysis of domestic and commercial technologies available on the market [6]. Figure 1 shows a simple structure of the HEGIT model, and Fig. 2 shows the input data, data processing and software integration used in the HEGIT framework. Three main features of the HEGIT models are:

• It incorporates an integrated unit commitment and capacity expansion planning problem for coordinated operation and long-term investment planning of the electricity and gas grids. This enables the model to assess different scenarios regarding the future of the gas grid and its role in providing low-carbon heating, quantify the system-wide implications of different pathways for decarbonising heating in buildings, identify the most efficient and effective use of available resources and technologies, identify strategies for maximising synergies between system planning goals and minimise trade-offs and provide technical evidence for policy making.
• It incorporates the flexible operation of heating technologies and demand response in coordinated planning of gas and electricity grids for decarbonising heating. This will allow the user to conduct simultaneous multi-scale system analysis for system flexibility planning and assess the potential benefits of access to a wider set of flexibility options through demand side management, and coordinate the planning of heating and electricity decarbonisation in order to avoid expansive stop-gap measures and long-term lock-in.
• It incorporates a multi-scale techno-economic representation of design features of heating technologies into whole energy system modelling and capacity planning (Fig. 3).

### HEGIT model scope and methodology

Models are usually tailored for a specific application or research question, which determines their complexity, modelling scope, and temporal and spatial resolutions [8]. Therefore, trade-off decisions should be made about these features to make the model computationally traceable. This section reviews the methodology, key features, main assumptions and input data in the HEGIT model. The HEGIT model is a least-cost optimisation (MILP) model and is designed to evaluate how different policies and decisions for decarbonising heat might impact the transformation and operation of electricity and gas grids over the planning horizon to 2050.

**Fig. 4:**
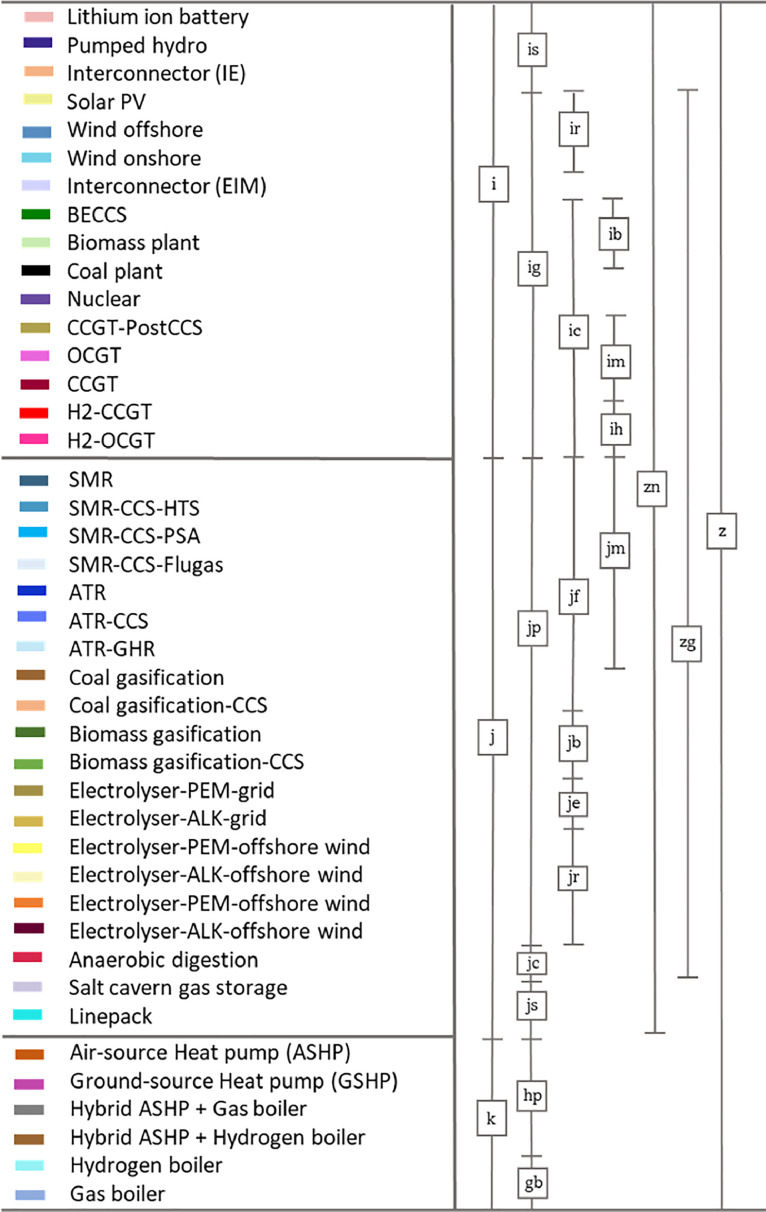
Types of technologies and their allocation to technology sets and subsets in the HEGIT model formulations. Please note that technology types can be adjusted or expanded.

*Modelling scope.* The ‘whole system’ scope includes all major gas and electric flows, detailed representations of technologies (including their flexibility attributes) for electricity generation and supply, gas production and supply, as well as decentralised heating generation technologies in buildings. Therefore, the model captures the interaction between electricity and gas grids and the heating in buildings through the conversion of energy vectors to each other and emission exchange/offsetting. Fig. 4 shows the list of technologies included in HEGIT mode. The high-level cost-optimisation process in the model analyses different combinations of technologies in each network and selects those that minimise the total system cost while meeting specified sustainability and security targets under technologies/networks’ operation and capacity expansion constraints.

*Long-term capacity planning.* HEGIT considers pathways from 2020 to 2050 by taking into account long-term trends in energy technologies, electricity and heating demands, fuel prices and carbon tax. The model considers annual time steps of 5 years over the planning horizon. The end of the planning horizon in the model can be modified based on the application and research questions.

*Temporal factors.* The energy system design is strongly influenced by temporal variations in demand and supply as well as peak levels of demand, especially as more variable renewable energy sources are integrated into the system. HEGIT accounts for seasonal and diurnal changes in demand and supply and explores the trade-offs between various ways to manage peak demand (e.g. supply, storage and demand-side measures). HEGIT has an adjustable temporal resolution. In order to reduce computational expenses, and because the full hourly version of the model takes a long time to solve, representative time steps can be used to reflect seasonal and diurnal variations in demand and supply. In our analysis, we use 13 representative days (12 representative days and one peak day), each with 24 consecutive hours. To identify the representative days for our analysis, we used k-means clustering with an energy-preserving approach^1^ proposed in Ref. [8].

*Spatial factors.* Geographic resolution is another important aspect to consider when designing energy systems, especially as distributed renewable resources are added to the system. the current version of the model represents a country as a single node, and the geographical distribution of resources and demand is not taken into account.

*Input data.* Large-scale bottom-up models typically require extensive data sets, which leads to a compromise between a highly disaggregated and detailed model on the one hand and data availability and model complexity on the other hand. The current version of HEGIT takes four types of data, as shown in Fig. 2:

• Temporal data: This includes demand profiles for heating and electricity, renewable energy sources availability, air and ground temperatures, and imported electricity prices.
• Technology data: This data includes capital investment, fixed operating costs, variable operating costs, and start-up costs, as well as the flexbility attributes and performance parameters such as economic lifetime, emission rate, efficiency, minimum safe operating level, uptime, downtime, ramp rates, inertia provision, and reserve provision for different technologies.
• System data: This includes resource price projections, resource availability data, discount rates, carbon tax projections, population data, number of households, emission budgets, as well as electricity and gas grids operating constraints.
• Heating technology data: This includes market cost and performance data on different domestic heating technologies, cost and performance data for various heat exchangers and compressors, thermodynamic parameters and specifications for different working fluids.

#### Assumption and simplifications

The other key assumptions considered in the model are:

• We used a central planner perspective, and our results do not take into account consumers’ preferences for heating technologies.
• Heating demand is population-adjusted, and detailed segmentation of housing stock is not considered in this version of the model.
• The regular electricity demand and the additional electricity demand from electrifying other sectors (such as transportation) are exogenously considered.
• Uncertainties in the input parameters are not considered, and the model is deterministic.
• A perfect market and perfect foresight over the planning horizon (2020–2050) is assumed.
• Energy efficiency improvements in buildings are assumed to be independent of the heating technology and vice versa.
• It is assumed that fuel switching occurs only if demand for heating is supplied by the direct burning of fossil fuels.
• To ensure the grid security for capacity planning and to take into account extreme events such as sustained periods of low wind and the risks from renewable sources’ intermittency; we assume that renewable sources will not be available on the peak day.

### Model formulation

As shown in Fig. 1, the HEGIT framework has four main parts: heating demand, heating technologies in buildings, gas and electricity grids and resources. In this section, we discuss the main constraints and assumptions for each part.^2^

#### Heating demand and fuel switching in buildings

The key constraint on the demand side in each planning year *a* is the balance between heating demand (both space heating and hot water) $q_{d}$ and the demand supplied by the conventional energy vector *v* in the existing system $q_{s}$ and the sum of demands supplied by alternative low-carbon energy vectors *l* that replace the energy vector *v*, $q_{lc}$, as shown in Eq. (1). For each conventional energy vector used for heating, the demand $d_{[}ev]$ is estimated using the average conversion factor λ (Eq. (3)). Eq. (4) indicates the constraint on the total direct annual emissions from heating in buildings at the consumer end.

(1) $$q_{s}\left(h,v,a\right)+\sum_{l}q_{lc}\left(l,h,v,a\right)=q_{d}\left(h,v,a\right)\;\;\;\forall \;h,v,a$$

(2) $$\Delta q_{s}\left(h,v,a\right)+\sum_{l}\Delta q_{lc}\left(l,h,v,a\right)=\delta_{eff}\left(h,v,a\right)\;\;\forall \;h,v,a$$

(3) $$d_{ev}\left(h,v,a\right)=q_{s}\left(h,v,a\right)\;/\;\lambda \left(h,v,a\right)\;\;\;\;\;\forall \;h,v,a$$

(4) $$\sum_{h,v}d_{ev}\left(h,v,a\right)\;CI\left(v,a\right)\leq Ems_{heat}\left(a\right)\;\;\;\;\forall \;a$$

The system is limited to only relying on electricity and gas grids and energy efficiency measures to decarbonise heat. Other options, such as solar thermal and heat networks, were not considered in our study since assessing the value of these distributed options requires a finer spatial resolution and regional energy system modelling. Furthermore, we assume that fuel switching occurs only if demand for heating is supplied by the direct burning of fossil fuels in individuation heating technologies in buildings. Consequently, if demand is already supplied by biomass, electricity or renewable sources, fuel switching does not occur (Eq. (5)).

(5) $$q_{lc}\left(l,h,nv,a\right)=0\;\;\;\;\;\;\;\;\;\;\;\;\forall \;nv\subset v,\;l=\left\{DH,ST\right\},h,a$$

A number of factors affect the demand for heating in buildings, including energy efficiency measures, household size, consumer behaviour, heating technology, *etc*. Thus, there are many uncertainties in estimating and projecting the heat demand profile and, more importantly, the peak demand for heating [9], [10], [11]. Our analysis of the transition in the heat sector is based on a whole-systems perspective, and heating demand is exogenously considered in our study.

**Fig. 5:**
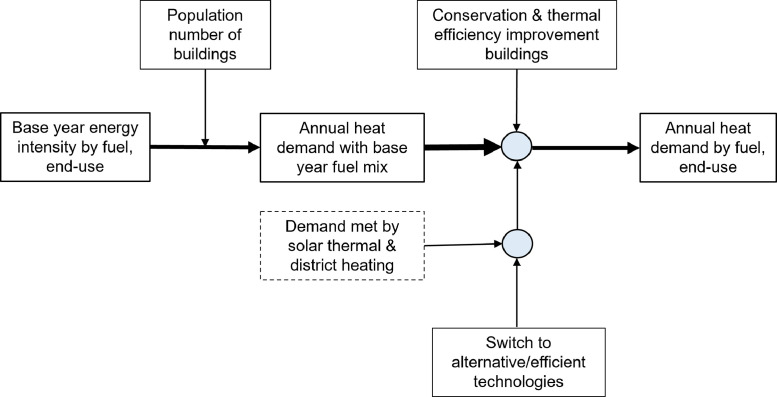
The methodology used to project heating demand over the planning horizon. The method was adopted from the contribution of Eyre et al. This graph is based on Fig. 2 in Ref. [10] with minor modifications.

The methodology shown in Fig. 5 (adopted with minor modifications from the contribution of Eyre et al. [10]) was used to project the heating demand over the planning horizon and create a baseline for heating demand. To account for energy efficiency uptake in buildings, we used average discount rates based on estimates reported in the literature [12], [13], [14] (Eq. (2)). Upon retrofitting energy efficiency measures into buildings, we assume the demand for heating will decline linearly by about 8% until 2030 and further by 22% until 2050. However, we do not take into account the impact of energy efficiency measures on heating demand profiles. We incorporated the building’s space heating and hot water demand profile for general cold weather as proposed by Sansom et al. [15]. Heating demand has been adjusted for population taking into account the changes in the population over the planning horizon in the UK as reported in Ref. [16]. Additionally, average household size was used to size the heating technologies [17], and detailed household segmentation was not considered. We differentiate, however, between gas grid-connected buildings and those not connected to the gas grid.

#### Heating technologies

Heating technologies in buildings convert the service demand for heating into demand for different energy vectors. Therefore, these technologies’ performance and operational features affect the operation and transition of both the gas and electricity networks and, ultimately, the cost of low-carbon energy carriers used for domestic heating. The HEGIT model implements five alternative low-carbon heating technologies to replace conventional fossil fuel boilers: air-source heat pump (ASHP) with electric backup, ground-source heat pump (GSHP) with electric backup, hybrid air-source heat pumps with natural gas boilers, hybrid air-source heat pumps with hydrogen boilers, and hydrogen boilers.

The main constraints for heating technologies are the heat demand balance as indicated in Eq. (6) and the energy and mass balances for the hot water cylinder and the heating technologies in each building, as shown in Eqs. (7) to (10). An electric or gas-fueled backup heater can be used for heat pumps depending on the scenario. For natural gas and hydrogen boilers, backup is not considered.

(6) $$q_{out}\left(k,a,c,t\right)+q_{backup}\left(k,a,c,t\right)=DP_{heat}\left(c,t\right)\;hd\left(k,a\right)\;\;\;\forall \;k,a,c,t$$

(7) $$q_{out}\left(k,a,c,t\right)=m_{out}\left(k,a,c,t\right)\;c_{p}\;\Delta T\;\;\;\;\;\;\forall \;k,a,c,t$$

(8) $$\Delta_{t}\;m_{tank}\left(k,a,c,t\right)=m_{in}\left(k,a,c,t\right)-m_{out}\left(k,a,c,t\right)\;\;\;\;\forall \;k,a,c,t$$

(9) $$m_{out}\left(k,a,c,t\right)\leq m_{tank}\left(k,a,c,t\right)\;\;\;\;\;\;\;\;\forall \;k,a,c,t$$

(10) $$m_{tank}\left(k,a,c,t\right)\leq \rho \;V_{tank}\left(k\right)\;\;\;\;\;\;\;\;\forall \;k,a,c,t$$

The output from each heat pump unit type *hp* at each time step *t* and day *c* is constrained by the coefficient of performance (COP) of the heat pump at that time step and its installed capacity (Eqs. (11) and (12)).

(11) $$COP\left(hp,c,t\right)\;p_{hp}\left(hp,a,c,t\right)=m_{in}\left(hp,a,c,t\right)\;c_{p}\;\Delta T\;\;\forall \;hp\subset k,a,c,t\;\;\;$$

(12) $$COP\left(hp,c,t\right)\;p_{hp}\left(hp,a,c,t\right)\leq NC_{HP}\left(a\right)\;\;\;\;\;\;\;\forall \;hp\subset k,a,c,t$$

For gas boilers, the thermal output is constrained by the installed capacity and the demand for gas (natural gas or hydrogen) is calculated based on the boiler’s efficiency, which is assumed to be the same for both hydrogen and gas boilers [18].

(13) $$\eta_{gb}\;d_{g}\left(gb,a,c,t\right)=\;m_{in}\left(gb,a,c,t\right)\;c_{p}\;\Delta T\;\;\;\;\;\;\forall \;gb\subset k,a,c,t$$

(14) $$m_{in}\left(gb,a,c,t\right)\;c_{p}\;\Delta T\leq NC_{gb}\;\;\;\;\;\;\;\;\;\;\;\forall \;gb\subset k,a,c,t$$

With respect to heat pumps, we use the empirical cost and performance equations from the integrated heat pump thermodynamic and component-costing model (discussed in Section “Introduction” and Fig. 2) to calculate the performance of heat pumps at each time step based on the outside air and ground temperatures^3^. For gas boilers single cost and performance estimations from market research and literature are used [[6], [19]]. As indicated in Eq. (15), the total consumers’ capital investment in fuel switching is calculated as the sum of the costs associated with purchasing heat pumps and their integrated hot water tanks, purchasing gas boilers, grid integration via smart meters, and installation costs. The costs of low-carbon alternative fuels are endogenously calculated in the model and therefore not included in the fuel switching cost.

(15) $$\begin{array}{cc} EUC & =\sum_{a}(\sum_{hp}b\left(hp,a\right)\;NC_{HP}\left(1+Ins\left(hp\right)\right)\;UC\left(hp\right)+\sum_{hp}b\left(hp,a\right)\;UC_{tank}\;V_{tank}\left(hp\right)\\ & \;+\sum_{hp}b\left(hp,a\right)\;UC_{SM}+\sum_{gb}b\left(gb,a\right)\;UC\left(gb\right)\;\left(1+Ins\left(gb\right)\right))/Disc\left(a\right) \end{array}$$

#### Gas and electricity grids

This part reviews the integrated capacity expansion planning and unit commitment optimisation problem in HEGIT that has been developed for modelling the coordinated operation and investment planning of the gas and electricity grids.

##### Gas network

Gas flow model nonlinearity (introduced by Weymouth gas equation) significantly impacts the computational time of the gas network models [20], [21], [22], [23]. One common approach to addressing this challenge is converting dynamic technical characteristics such as pressure and volume into a static energy unit, which is our preferred approach for HEGIT. With this simplification a linear model of the gas grid can be developed [24], [25], [26]. This section reviews the problem definition and main constraints considered for modelling the gas network.

*System-wide constraints*. We assume that two different types of gases can be injected into the gas grid: methane and hydrogen. The main uses of methane in the electricity and heating sectors are for gas-fired power plants, hydrogen production using methane reforming, the direct use of methane in gas boilers in buildings and other demand from other sectors such as industry (shown in Eq. (73)). Equation (16) shows the demand/supply balance for methane for heating and power, which can be supplied either by natural gas (from domestic production and import) or biomethane.

(16) $$\begin{array}{cc} d_{NG}\left(a,c,t\right)+d_{BM}\left(a,c,t\right) & =\sum_{h}d_{ev}\left(h,v,a\right)DP_{heat}\left(c,t\right)+\sum_{im}\;p_{e}\left(im,a,c,t\right)/\gamma \left(im\right)\\ & \;+\sum_{jm}\;p_{H_{2}}\left(jm,a,c,t\right)/\gamma \left(jm\right)\;\forall \;v=gas,\;a,c,t\;\;\; \end{array}$$

Demand for hydrogen in each planning year is calculated based on the decarbonisation pathway adopted for heating as well as the system-wide and operational constraints in each scenario (Eq. (17)). In the hydrogen network, Eq. (18) ensures the hydrogen demand and supply balance at each time step *t*, representative day *c* in the planning year *a*. Equation (19) denotes the operating and supply margin requirements (to account for 1 in 20 demand (*OM* coefficient) and N−1 condition (*SM* coefficient) [27]) in the hydrogen network. Hydrogen import is not taken into account in the current version of the model, and it was assumed that all hydrogen would be produced domestically [28]. Therefore hydrogen storage facilities, linepacks, and production flexibility are the only mechanisms we considered for operating margin provision.

(17) $$\sum_{c,t}\eta_{gb}\;d_{g}\left(H_{2}boiler,a,c,t\right)\;WF\left(c\right)\geq \sum_{h,v}q_{lc}\left(l,h,v,a\right)\;\forall \;a,l=H_{2}$$

(18) $$\sum_{jp}pd_{H_{2}}\left(jp,a,c,t\right)+\sum_{js}sd_{H_{2}}\left(js,a,c,t\right)=d_{g}\left(H_{2}boiler,a,c,t\right)\left(1+NL\right)-ug_{H_{2}}\left(a,c,t\right)\;\forall a,c,t$$

(19) $$\sum_{jf}x\left(jf,a\right)\;NC\left(jf\right)\;TF\left(jf,P_{max}\right)\;+LP_{Max}\;d_{H_{2}}\left(a\right)+\sum_{jus}x\left(jus,a\right)\;F_{outMax}\left(jus\right)\geq PL_{H_{2}}\left(a\right)\;\left(1+OM\right)+SM\;\forall a$$

*Gas production plants operation:*Hydrogen production units’ output can be used to meet demand or to charge storage units, including linepack (Eqs. (20) and (21)). The hydrogen output from production units is limited by their capacity, and in the case of renewable hydrogen production units, the output is also limited by the availability of renewable resources (Eqs. (22) to (25)). The same equations apply to biomethane production units. To avoid repetition we haven’t listed them here.

(20) $$pd_{H_{2}}\left(jp,a,c,t\right)+pst_{H_{2}}\left(jp,a,c,t\right)=p_{H_{2}}\left(jp,a,c,t\right)\;\forall jp,a,c,t$$

(21) $$\sum_{jp}pst_{H_{2}}\left(jp,a,c,t\right)=\sum_{js}ps_{H_{2}}\left(js,a,c,t\right)\;\forall a,c,t$$

(22) $$p_{H_{2}}\left(jp,a,c,t\right)\leq n\left(jp,a,c,t\right)\;NC\left(jp\right)\;\forall \;jp,a,c,t$$

(23) $$p_{H_{2}}\left(jp,a,c,t\right)\geq n\left(jp,a,c,t\right)\;TF\left(jp,P_{min}\right)\;NC\left(jp\right)\;\forall \;jp,a,c,t$$

(24) $$p_{H_{2}}\left(jr,a,c,t\right)\geq n\left(jr,a,c,t\right)\;TF\left(jr,P_{min}\right)\;NC\left(jr\right)\;AV\left(jr,c,t\right)\;\forall \;jr,a,c,t$$

(25) $$p_{H_{2}}\left(jr,a,c,t\right)\leq n\left(jr,a,c,t\right)\;NC\left(jr\right)\;AV\left(jr,c,t\right)\;\forall \;jr,a,c,t$$

Equation (26) represents the electricity demand from the grid-integrated electrolyser units at each time step:

(26) $$p4H_{2}\left(a,c,t\right)=\sum_{je}p_{H_{2}}\left(je,a,c,t\right)/\gamma \left(je\right)\;\forall \;a,c,t$$

*Gas storage units operation*. Equation (27) to (40) represents the operational constraints of underground gas storage units. The inventory balance, the minimum and maximum storage capacity of each storage unit are described by Eqs. (27), (28) and (29) respectively. Equations (30) to (34) constrains the injection and withdrawal rates from storage units. Constraint (35) ensures sufficient reserve hydrogen storage to meet minimum days of average hydrogen demand, represented by the parameter *Res*.

(27) $$\Delta_{t}s_{H_{2}}\left(jus,a,c,t\right)=\left(ps_{H_{2}}\left(jus,a,c,t\right)\;\mu \left(jus\right)\right)-sd_{H_{2}}\left(jus,a,c,t\right)-stl_{H_{2}}\left(jus,a,c,t\right)\;\forall \;jus,a,c,t$$

(28) $$s_{H_{2}}\left(jus,a,c,t\right)\geq o\left(jus,a,c,t\right)\;S_{Cush}\left(jus\right)\;NC_{s}\left(jus\right)\;\forall \;jus,a,c,t$$

(29) $$s_{H_{2}}\left(jus,a,c,t\right)\leq o\left(jus,a,c,t\right)\;NC_{s}\left(jus\right)\;\forall \;jus,a,c,t$$

(30) $$ps_{H_{2}}\left(jus,a,c,t\right)\leq o\left(jus,a,c,t\right)\;F_{inMax}\left(jus\right)\;\forall \;jus,a,c,t$$

(31) $$sd_{H_{2}}\left(jus,a,c,t\right)+stl_{H_{2}}\left(jus,a,c,t\right)\leq o\left(jus,a,c,t\right)\;F_{outMax}\left(jus\right)\;\forall \;jus,a,c,t$$

(32) $$sd_{H_{2}}\left(jus,a,c,t\right)+stl_{H_{2}}\left(jus,a,c,t\right)\leq s_{H_{2}}\left(jus,a,c,t\right)\;\mu \left(jus\right)\;\forall \;jus,a,c,t$$

(33) $$F_{outMax}\left(jus\right)\leq WD\;\left(s_{H_{2}}\left(jus,a,c,t\right)-S_{Cush}\left(jus\right)\;NC_{s}\left(jus\right)\right)\;\forall \;jus,a,c,t$$

(34) $$F_{inMax}\left(jus\right)\leq INJ\;\left(s_{H_{2}}\left(jus,a,c,t\right)-S_{Cush}\left(jus\right)\;NC_{s}\left(jus\right)\right)\;\forall \;jus,a,c,t$$

(35) $$\sum_{\mathrm{jus}}s_{H_{2}}\left(\mathrm{jus},a,c,t\right)\geq \mathrm{Res}\;\bar{d_{H_{2}}}\left(a\right)\;+\sum_{\mathrm{jus}}\;x\left(\mathrm{jus},a\right)\;S_{\mathrm{Cush}}\left(\mathrm{jus}\right)\;NC_{s}\left(\mathrm{jus}\right)\;\;\forall a,c,t$$

(36) $$x\left(jus,a\right)\;NC_{s}\left(jus\right)\leq Cap_{UK}\left(jus\right)\;\forall \;jus,a$$

In the current version of the model, we only consider underground gas storage in salt caverns^4^ for bulk hydrogen storage. Nonetheless, we distinguish between different types of salt caverns based on the rock formation and depth of the caverns. Prospective areas and sites are, however, constrained by the presence of salt beds, the depth and salt thickness required for the safe design, construction and operation of caverns, as well as their proximity to demand clusters [29]. The cushion gas^5^ capacity of all caverns is considered to be 30% of the storage capacity of the cavern. Investment and operating costs, the capacity of each cavern, the maximum available capacity for each formation, and their operating characteristics for the case study of the UK are gathered from the following Ref. [30], [29], [31], [32], [33], [34], [35]. Furthermore, we used the maximum injection and withdrawal rates based on expert interviews^6^, which are consistent with the figures reported by ETI [32] and Ref. [30].

The calculation of the available linepack^7^ within the gas network is complex and requires combining spatial modelling of gas demand with spatial modelling of the gas transmission and distribution network. Since in the current version of HEGIT, we use a single node representation of the electricity and gas networks in the UK, and to simplify the analysis and avoid nonlinearities of gas flow equations, we did not incorporate the gas flow through the pipeline into the current version of the model. Instead, we use the approach for rating different pipelines’ capacities, proposed by Vega et al. [25] to estimate the available linepack in the gas network. Thus, the effective linepack within the gas network was modelled as a storage technology (the balances were calculated in static energy units instead of volume). In the steady-state condition, linepack within a pipeline is proportional to average pressure along it [36]. Using the gas equation of state and Boyle’s law [[36], [37]], the linepack’s maximum and minimum state of charge was calculated using maximum and minimum pressure limits, length and diameter of different gas distribution and transmission pipelines, and the calorific value of gases, based on the UK’s gas network data available in References [[38], [39], [40]]. The balance of the hydrogen stored as linepack in the pipelines is represented in Eqs. (37) to (39). Equation (39) ensures the balance of the gas stored as a linepack in a gas day. The linepack capacity is constrained by the degree of conversion of the gas grid to hydrogen as denoted in Eq. (40).

(37) $$\Delta_{t}s_{H_{2}}\left(jl,a,c,t\right)=ps_{H_{2}}\left(jl,a,c,t\right)+sl_{H_{2}}\left(jl,a,c,t\right)-sd_{H_{2}}\left(jl,a,c,t\right)\;\forall \;jl,a,c,t$$

(38) $$sd_{H_{2}}\left(jl,a,c,t\right)\leq s_{H_{2}}\left(jl,a,c,t\right)\;\forall \;a,c,t$$

(39) $$\sum_{t}ps_{H_{2}}\left(jl,a,c,t\right)+sl_{H_{2}}\left(jl,a,c,t\right)-sd_{H_{2}}\left(jl,a,c,t\right)=0\;\forall \;jl,a,c$$

(40) $$LP_{Min}\;d_{H_{2}}\left(a\right)\leq s_{H_{2}}\left(jl,a,c,t\right)\leq LP_{Max}\;d_{H_{2}}\left(a\right)\;\forall \;jl,a,c,t$$

##### Electricity grid

*Electricity system security constraints*. The unit commitment problem for the electricity system is built upon the modelling contribution of Heuberger [1] with incorporating insights from Tkiouat et al., Shahidehpour et al. and Ameli et al. [[20], [22], [41]]. For each time *t*, day *c* and year *a*, the total electricity demand is calculated by summing the regular electricity demand from different sectors, demand from already electrified heat in both buildings and industry, projected electricity demand from EVs uptake, additional electricity required for electrifying heating, and additional electricity demand from the gas grid as described in Eq. (41).

(41) $$\begin{array}{cc} d_{e}\left(a,c,t\right) & =\sum_{hp}\left(p_{hp}\left(hp,a,c,t\right)+p_{bh}\left(hp,a,c,t\right)\right)+DP_{EV}\left(c,t\right)\;D_{EV}\left(a\right)+p4H_{2}\left(a,c,t\right)+p4gs\left(a,c,t\right)\\ & \;+DP_{elec}\left(c,t\right)\;\left(D_{elec}\left(a\right)+\sum_{h}\frac{\sum_{a^{\prime }}\delta_{eff}\left(h,electricity,a^{\prime }\right)}{\lambda_{h,electricity,a}}\right)\;\forall a,c,t\;\;a^{\prime }\leq a \end{array}$$

The electricity system security constraints are represented in Eqs. (42) to (45). Equation (42) represents the balance between the electricity demand and supply from generation units *ig* and storage units *is* at each time step over the planning horizon. Equations (43) and (44) ensure the capacity margin and reserve margin in the system (set based on regional/local grid regulations) and the reserve buffer requirements to make up for the intermittency in renewable generators’ output [41]. Equation (45) denotes the minimum level of inertia required in the electricity grid at every time step (set based on N-1 rule for each year).

(42) $$\sum_{ig}pd_{e}\left(ig,a,c,t\right)+\sum_{is}sd_{e}\left(is,a,c,t\right)=d_{e}\left(a,c,t\right)\;\left(1+TL\right)-up_{d}\left(a,c,t\right)\;\forall \;a,c,t$$

(43) $$\sum_{i}x\left(i,a\right)\;NC\left(i\right)\;TE\left(i,Cmax\right)=PL_{e}\left(a\right)\;\left(1+CM\right)\;\forall \;a,c,t$$

(44) $$\sum_{ig}r_{e}\left(ig,a,c,t\right)\;TE\left(ig,Rp\right)+\sum_{is}sr_{e}\left(is,a,c,t\right)\;TE\left(is,Rp\right)\geq PL\left(a\right)\;RM+\sum_{ir}pd_{e}\left(ir,a,c,t\right)\;WR\;\forall \;a,c,t$$

(45) $$\sum_{\mathrm{ig}}n\left(\mathrm{ig},a,c,t\right)\;\mathrm{NC}\left(\mathrm{ig}\right)\;\mathrm{TE}\left(\mathrm{ig},\mathrm{Ip}\right)+\sum_{\mathrm{is}}o\left(\mathrm{is},a,c,t\right)\;\mathrm{NC}\left(\mathrm{is}\right)\;\mathrm{TE}\left(\mathrm{is},\mathrm{Ip}\right)\geq \mathrm{SI}\left(a\right)\;\forall \;a,c,t$$

*Electricity generating plants operation*. Each generation unit would either directly supply electricity demand or charge grid-integrated storage units as denoted in Eq. (46).

(46) $$pd_{e}\left(ig,a,c,t\right)+ps_{e}\left(ig,a,c,t\right)=p_{e}\left(ig,a,c,t\right)\;\forall \;ig,a,c,t$$

The electricity output from thermal plants is bounded between the minimum and maximum limits of units’ capacity (Eqs. (47) and (48)), and in the case of renewable generation units the upper and lower bounds are determined by the availability of the renewable sources at each time step (Eqs. (49) and (50)). Equation (51) denotes a constraint on the total annual emission from electricity generation.

(47) $$p_{e}\left(ig,a,c,t\right)+r_{e}\left(ig,a,c,t\right)\leq n\left(ig,a,c,t\right)\;NC\left(ig\right)\;TE\left(ig,Pmax\right)\;\forall \;ig,a,c,t$$

(48) $$p_{e}\left(ic,a,c,t\right)\geq n\left(ic,a,c,t\right)\;NC\left(ic\right)\;TE\left(ic,Pmin\right)\;\forall \;ic,a,c,t$$

(49) $$p_{e}\left(ir,a,c,t\right)\geq n\left(ir,a,c,t\right)\;NC\left(ir\right)\;TE\left(ir,Pmin\right)\;AV(ir,c,t)\;\forall \;ir,a,c,t$$

(50) $$p_{e}\left(ir,a,c,t\right)\leq n\left(ir,a,c,t\right)\;NC\left(ir\right)\;AV\left(ir,c,t\right)\;\forall \;ir,a,c,t$$

(51) $$\sum_{ig,c,t}p_{e}\left(ig,a,c,t\right)\;WF\left(c\right)TE\left(ig,Ems\right)\leq SE\left(a\right)\;\forall \;a$$

*Electricity storage unit operation*. Equations (52) to (57) describes the operation constraints for grid-connected electricity storage units. Equation (52) shows the inventory balance of storage units at each time step. The level of storage of each unit is bounded by its maximum and minimum states of charge, as given in Eq. (53). Equations (54) to (57) represent the charging and discharging constraints of storage units.

(52) $$\Delta_{t}\;s_{e}\left(is,a,c,t\right)=pis_{e}\left(is,a,c,t\right)\;\mu \left(is\right)-sd_{e}\left(is,a,c,t\right)\;\forall \;is,a,c,t$$

(53) $$o\left(is,a,c,t-1\right)\;S_{Min}\left(is\right)\leq s_{e}\left(is,a,c,t\right)\leq o\left(is,a,c,t-1\right)S_{Max}\left(is\right)\;\forall \;is,a,c,t$$

(54) $$sd_{e}\left(is,a,c,t\right)+sr_{e}\left(is,a,c,t\right)\geq o\left(is,a,c,t\right)\;NC\left(is\right)\;TE\left(is,Pmin\right)\;\forall \;is,a,c,t$$

(55) $$sd_{e}\left(is,a,c,t\right)+sr_{e}\left(is,a,c,t\right)\leq o\left(is,a,c,t\right)\;NC\left(is\right)\;\forall \;is,a,c,t$$

(56) $$sd_{e}\left(is,a,c,t\right)+sr_{e}\left(is,a,c,t\right)\leq s_{e}\left(is,a,c,t\right)\;\mu \left(is\right)\;\forall \;is,a,c,t$$

(57) $$pis_{e}\left(is,a,c,t\right)\leq o\left(is,a,c,t\right)\;NC\left(is\right)\;\forall \;is,a,c,t$$

##### Up time Down time constraints

For all the generating units in both electricity and gas networks *zg*, the constraints on the state of operation is given in the Eqs. (58) to (63). Please note that separate variables are used for units that are shutting down *w* or starting up *u* as shown in Eqs. (58) to (61). Equations (62) to (63) shows the ramping constraints for different generation units.

(58) $$u\left(zg,a,c,t\right)\geq \Delta_{t}n\left(zg,a,c,t\right)\;\forall \;zg,a,c,t$$

(59) $$w\left(zg,a,c,t\right)\geq -\Delta_{t}n\left(zg,a,c,t\right)\;\forall \;zg,a,c,t$$

(60) $$u\left(zg,a,c,t\right)\leq n\left(zg,a,c,\tau \right)\;\forall \;zg,a,c,\tau =t+t^{\prime }-1,\;\;t^{\prime }\leq UT\left(zg\right)$$

(61) $$w\left(zg,a,c,t\right)\leq x\left(zg,a\right)-n\left(zg,a,c,\tau \right)\;\forall \;zg,a,c,\tau =t+t^{\prime }-1,\;\;t^{\prime }\leq DT\left(zg\right)$$

(62) $$\Delta_{t}\;p_{H_{2},e,BM}\left(zg,a,c,t\right)\leq n\left(zg,a,c,t\right)\;NC\left(zg\right)\;RU\left(zg\right)\;\forall \;zg,a,c,t$$

(63) $$\Delta_{t}\;p_{H_{2},e,BM}\left(zg,a,c,t\right)\geq -n\left(zg,a,c,t\right)\;NC\left(zg\right)\;RD\left(zg\right)\;\forall \;zg,a,c,t$$

##### Capacity planning and system expansion

For all the technologies in the electricity and gas networks and the heating technologies *z*, capacity balances over the planning horizon are represented in Eqs. (64) to (68), considering technologies’ economic lifetime.

(64) $$x\left(z,a\right)=X_{ini}\left(z\right)\;\forall \;z,a=1$$

(65) b(z,a)≤Br(z)Δa∀z,a

(66) $$x\left(z,a\right)=x\left(z,a-1\right)+b\left(z,a-\frac{LT_{ini}\left(i\right)}{\Delta a}\right)+b\left(z,a\right)\;\forall \;\;z,a\leq \frac{LT_{ini}\left(z\right)}{\Delta a}+1$$

(67) $$x\left(z,a\right)=x\left(z,a-1\right)+b\left(z,a\right)\;\forall \;\;z,\frac{LT_{ini}\left(z\right)}{\Delta a}+1<a\leq \frac{LT(z)}{\Delta a}+1$$

(68) $$x\left(z,a\right)=x\left(z,a-1\right)+b\left(z,a-\frac{LT(z)}{\Delta a}\right)+b\left(z,a\right)\;\forall \;\;z,a>\frac{LT(z)}{\Delta a}+1$$

#### Resource availability

The resource part takes into account the supply curve and availability of different primary resources. To account for the availability of biomass as one of the controversial and critical resources for meeting the net-zero emission target, and also to take into account the cost variations for different types and sources of biomass, the biomass supply curves for the UK proposed by Zhang et al. and S2Biom data set [[42], [43]] is incorporated into the model as given in Eqs. (69) to (72).

(69) $$d_{bms}\left(a\right)=\sum_{h}d_{ev}\left(biomass,h,a\right)+\sum_{ib,c,t}p_{e}\left(ib,a,c,t\right)\;WF\left(c\right)/\gamma \left(ib\right)+\sum_{ib,c,t}p_{H_{2}}(jb,a,c,t)\;WF(c)/\gamma (jb)\;\forall \;a$$

(70) $$su_{bms}\left(bt,a\right)\leq Max_{bms}\left(bt\right)\;\forall \;a,bt$$

(71) $$\sum_{bt}su_{bms}\left(bt,a\right)=d_{bms}\left(a\right)\;\forall \;a$$

(72) $$\sum_{bt}su_{bms}\left(bt,a\right)Pr_{bms}\left(bt\right)=cost_{bms}\left(a\right)\;\forall \;a$$

The sources of supplying natural gas include imports and domestic production (Eq. (73)). However, no constraint is considered on the availability of fossil fuel resources (coal, oil and natural gas imports).

(73) $$\sum_{c,t}d_{NG}\left(a,c,t\right)\;WF\left(c\right)+IO_{NG}\left(a\right)\leq P_{NG}\left(a\right)+imp_{NG}\left(a\right)\;\forall a$$

The availability of renewable sources are incorporated by parameter *AV* in Eqs. (24) to (25) and (49) to (50). Data on wind and solar availability is obtained from the Renewable Ninja website.

#### Model objective function and key performance indicators

The objective function of the model is the total system transition cost. The total system cost is calculated as the sum of the capital investment required the electricity and gas networks, cost of fuel switching at the consumer side and operating costs over the planning horizon (Eq. (74)). The operating cost takes into account the ongoing cost of generation units including resource costs, startup and shutdown cost, emission costs, and the cost of loss of load (Eq. (77)). The total operating cost over the planning horizon is calculated using the Trapezoidal rule [44] of integration as represented in Eq. (75) considering annual time steps of 5 years.^8^

(74) $$\begin{array}{cc} TSC & =\sum_{zn,a}b\left(zn,a\right)\;WFA\left(zn\right)\;CAPEX\left(zn\right)\;NC\left(zn\right)\;NRF/Disc\left(a\right))+\int_{a}OPEX\left(a\right)\;da\\ & +\underset{l=electricity}{\sum_{\;\;\;\;a,h,v=gas}C_{dcg}}\;\left(\Delta_{a}\;q_{lc}\left(l,h,v,a\right)/\lambda \left(h,v,a\right)\right)/Disc\left(a\right)+EUC \end{array}$$

(75) $$\int_{a}OPEX\left(a\right)\;da=\frac{1}{2}\;\Delta \;a\;\left[OPEX\left(a_{1}\right)+OPEX\left(a_{n}\right)+2\;\sum_{a_{2}}^{a_{n-1}}OPEX\left(a\right)\right]$$

The total system emission is also calculated the sum of emissions from the electricity, gas and heat systems as presented bellow:

(76) $$\begin{array}{cc} TSE & =\int_{a}(\sum_{ig,c,t}p_{e}\left(ig,a,c,t\right)\;WF\left(c\right)\;TE\left(ig,Ems\right)+\sum_{jp,c,t}p_{H_{2}}\left(jp,a,c,t\right)\;WF\left(c\right)\;TF\left(jp,Ems\right)\\ & \;+\sum_{h,v}d_{ev}\left(h,v,a\right)\;CI\left(v,a\right)+\sum_{jc,c,t}p_{BM}\left(jc,c,t\right)\;WF\left(c\right)\;e_{BM}\left(jc\right))\;da\; \end{array}$$

(77) $$\begin{array}{cc} OPEX(a) & =(\sum_{ig,c,t}u\left(ig,a,c,t\right)\;WF\left(c\right)\;OPEXSU\left(ig\right)\\ & \;+\sum_{ig,c,t}\left(p_{e}\left(ig,a,c,t\right)\;WF\left(c\right)\;OPC\left(ig,a\right)+n\left(ig,a,c,t\right)\;WF\left(c\right)\;OPEXF\left(ig\right)\right)\\ & \;+\sum_{ic,c,t}p_{e}\left(ic,a,c,t\right)\;WF\left(c\right)\;Ctx\left(a\right)\;TE\left(ic,Ems\right)\\ & \;+\sum_{is,c,t}\left(sd_{e}\left(is,a,c,t\right)\;WF\left(c\right)\;OPC\left(is,a\right)+o\left(is,a,c,t\right)\;WF\left(c\right)\;OPEXF\left(is\right)\right)\\ & \;+\sum_{c,t}p_{e}\left(InterImp,a,c,t\right)\;WF\left(c\right)\;pr_{impElec}+\sum_{c,t}up_{d}\left(a,c,t\right)\;WF\left(c\right)\;VoLL_{e}\\ & \;+\sum_{jp,c,t}u\left(jp,a,c,t\right)\;WF\left(c\right)\;OPEXSU\left(jp\right)\\ & \;+\sum_{jp,c,t}\left(p_{H_{2}}\left(jp,a,c,t\right)\;WF\left(c\right)\;OPC\left(jp,a\right)+n\left(jp,a,c,t\right)\;WF\left(c\right)\;OPEXF\left(jp\right)\right)\\ & \;+\sum_{jp,c,t}p_{H_{2}}\left(jp,a,c,t\right)\;WF\left(c\right)\;Ctx\left(a\right)\;TF\left(jp,Ems\right)\\ & \;+\sum_{js,c,t}\left(sd_{H_{2}}\left(js,a,c,t\right)\;WF\left(c\right)\;OPC\left(js,a\right)+o\left(js,a,c,t\right)\;WF\left(c\right)\;OPEXF\left(js\right)\right)\\ & \;+\sum_{jp,c,t}p_{H_{2}}\left(jp,a,c,t\right)\;WF\left(c\right)\;Ctx\left(a\right)\;TF\left(jp,Ems\right)\\ & \;+\sum_{c,t}ug_{H_{2}}\left(a,c,t\right)\;WF\left(c\right)\;VoLL_{g}+\sum_{c,t}ug_{CH_{4}}\left(a,c,t\right)\;WF\left(c\right)\;VoLL_{g}\\ & \;+\sum_{jc,c,t}\left(p_{BM}\left(jc,a,c,t\right)\;WF\left(c\right)\;OPC\left(jc,a\right)+n\left(jc,a,c,t\right)\;WF\left(c\right)\;OPEXF\left(jc\right)\right)\\ & \;+\sum_{jc,c,t}p_{BM}\left(jc,a,c,t\right)\;WF\left(c\right)\;Ctx\left(a\right)\;e_{BM}\left(jc\right)\\ & \;+\sum_{h,rv}d_{ev}\left(h,rv,a\right)\;Pr_{ev}\left(rv\right)\;+\;cost_{bms}\left(a\right)\\ & \;+\sum_{h,v}d_{ev}\left(h,v,a\right)\;CI\left(v,a\right)\;Ctx\left(a\right))/Disc\left(a\right)\;\forall \;a \end{array}$$

*Cost of avoided*${\text{CO}}_{2}$*emissions* Cost of avoided ${\text{CO}}_{2}$ is an indicator of the costs incurred in the system for each tonne of ${\text{CO}}_{2}$ emission avoided. It is calculated as the ratio of the difference in total system cost in a low carbon scenario (here we considered the net-zero emission scenarios (Net0)) and the business as usual scenario (BAU) to the total avoided emissions, as represented in Eq. (78). In the BAU scenario, there are no emissions mitigation targets for heating in buildings and the transition of the electricity and gas grids is driven by changes in demand and electricity grid decarbonisation targets.

(78) $$CAC(£/t_{{\mathrm{CO}}_{2}})=\frac{TSC_{Net0}-TSC_{BAU}}{TSE_{BAU}-TSE_{Net0}}$$

*Shannon Weiner Diversity Index (SWDI)*. SWDI is a quantitative measure that reflects the diversity of the members of a set and is widely used in long-term energy planning and security of supply studies. Higher index values indicate a more diverse mix [[45], [46]]. According to Jenson et al. it is the best single indicator of diversity for long-term energy supply security as it reflects on both variety and balance of categories [46]. Equation (79) shows the general equation for the Shannon index where y(n) is the proportion of each category. In this study, we use the Shannon index to evaluate the diversity of the primary resources used for supplying electricity and heating.

(79) $$SWDI=-\sum_{n}y\left(n\right)\;\ln y\left(n\right)$$

### CRediT authorship contribution statement

**Pooya Hoseinpoori:** Conceptualization, Methodology, Software, Formal analysis, Validation, Data curation, Visualization, Writing – original draft. **Jeremy Woods:** Supervision, Writing – review & editing. **Nilay Shah:** Supervision, Methodology, Writing – review & editing.

## Declaration of Competing Interest

The authors declare that they have no known competing financial interests or personal relationships that could have appeared to influence the work reported in this paper.

## Data Availability

The data used for this model is available as supplementary data in the related research paper: https://doi.org/10.1016/j.enconman.2022.115952.

## Footnotes

1 The code is developed in R, and further information about details of this approach are available in Reference [8].

2 The constraints are presented in their general form. Some of the equations may vary for different scenarios.

3 Please check Ref. [7]. for examples of these cost and performance curve equations.

4 Salt caverns are commonly used for large-scale gas storage. In the UK, the caverns at Tees Valley have been used for hydrogen storage in the past. Although depleted hydrocarbons (oil or gas) fields and aquifers can also be used for storing gas, they are not considered suitable for storing hydrogen due to residual contaminants, such as sulphur compounds and hydrocarbons, high leakage rates, low operating pressure ranges *etc.* [30].

5 Salt caverns require different cushion gas requirements depending on the site characteristics. The amount of cushion gas required is determined by the minimum pressure required to prevent salt creep that damages cavern integrity. Generally, about one-third of the cavern volume will contain cushion gas if the salt cavern is operated under variable pressures [31]

6 “Cavern filling and emptying rates are governed by the maximum flow rates in the boreholes and the maximum pressure reduction rates in the caverns, which are determined by local rock geomechanical properties and operational requirements. In practice, each proposed cavern development should be subjected to detailed geomechanical modelling studies to assess its operational limits. For gas storage in general, the rate is 0.8-1 MPa/day, with maximum withdrawal rates of 10% of the working gas per day and injection rates of 2–3% of the working gas per day”.

7 According to National Grid,“The amount of gas within the gas network at any time is known as ‘linepack’. The acceptable range over which the amount of gas in the network can vary and the ability to further compress and expand this gas is generally referred to as ‘linepack flexibility”’ [47].

8 The cost of reinforcing electricity transmission and distribution networks is assumed to add respectively 7% and 4% of the total capital investment and cost of conversion of the gas network and building new hydrogen transmission pipelines is assumed to add 13% of the total capital investment to the total system cost [[48], [49], [50]].
